# Bioequivalence and safety evaluation of two preparations of metformin hydrochloride sustained-release tablets (Boke^®^ and Glucophage^®^-XR) in healthy Chinese volunteers: a randomized phase I clinical trial

**DOI:** 10.1080/07853890.2022.2125574

**Published:** 2022-09-22

**Authors:** Ming-Li Sun, Chen Liu, Hai-Hong Bai, Ya-Li Wei, Wei Zhang, Hui-Juan Liu, Yin-Juan Li, Long Liu, Yu Wang, Yuan-Xv Tong, Qian Gao, Qian-Ying Liu, Xinghe Wang

**Affiliations:** a Phase I Clinical Trial Centre, Beijing Shijitan Hospital Affiliated to Capital Medical University, Beijing, P. R. China; b Division of Medicine, China Resources Double-Crane Pharmaceutical Co. Ltd., Beijing, P. R. China; c Research and Development Department, China Resources Double-Crane Pharmaceutical Co. Ltd., Beijing, P. R. China

**Keywords:** Bioequivalence, crossover trials, metformin hydrochloride, pharmacokinetics, fasting conditions

## Abstract

**Background:**

As per the National Medical Products Administration (NMPA) requirements, the quality and efficacy of generic drugs must be consistent with those of the innovator drug. We aimed to evaluate the bioequivalence and safety of generic metformin hydrochloride sustained-release (MH-SR) tablets (Boke^®^) developed by Beijing Wanhui Double-crane Pharmaceutical Co. Ltd., China and the innovator product metformin hydrochloride extended-release tablets (Glucophage^®^-XR) manufactured by Bristol-Myers Squibb Company, New York, NY, in healthy Chinese volunteers.

**Materials and methods:**

We performed a bioequivalence and safety assessment of MH-SR (500 mg/tablet) and Glucophage^®^-XR (500 mg/tablet) tablets in a randomized, open-label, two-period, two-sequence crossover, single-dose oral study in 48 healthy Chinese adult participants under fasting conditions (Chinese Clinical Trial Registration No. CTR20171306). The washout period was seven days. Bioequivalence (80.00–125.00%) was assessed using adjusted geometric mean ratios (GMRs) and two-sided 90% confidence intervals (CIs) of the area under the curve (AUC) and maximum concentration (*C*_max_) for each component.

**Results:**

The 90% CIs of the test/reference preparation for key pharmacokinetic parameters were 97.36–108.30% for AUC_0→_*_t_*, 97.26–108.09% for AUC_0→∞_ and 96.76–111.37% for *C*_max_. No severe adverse events (AEs) were observed. However, 38 adverse drug reactions (ADRs) occurred, including metabolic or nutritional conditions (*n* = 8), infections (*n* = 2), gastrointestinal conditions (*n* = 10) and abnormal inspection (*n* = 18). No significant difference was observed between MH-SR (23 ADRs, 10 participants) and Glucophage^®^-XR (15 ADRs, 12 participants) (*p* = .500). Bioequivalence was concluded since the 90% CIs of the main pharmacokinetic parameters were within the equivalence interval (80.00–125.00%).

**Conclusions:**

MH-SR (500 mg/tablet) and Glucophage^®^-XR (500 mg/tablet) were found to be bioequivalent and safe under fasting conditions in healthy Chinese participants. Thus, the market demand for MH-SR tablets (500 mg/tablet) can be met using the generic alternative.

**KEY MESSAGES**
- Generic MH-SR tablets (500 mg, Beijing Wanhui Double-crane Pharmaceutical Co. Ltd., Beijing, China) and innovator MH-SR tablets (Glucophage^®^-XR, 500 mg, Bristol-Myers Squibb Company, New York, NY, USA) were bioequivalent and safe in healthy Chinese volunteers under single-dose administration and fasting conditions.
- The main goal of this study is to support an increase in the supply of MH-SR tablets in China by proving the efficacy and safety of a generic alternative.
- Although no sugar was administered in the BE trial of the MH-SR tablets under fasting conditions, no hypoglycaemic event occurred. The method used in this study is expected to serve as a reference for BE studies of different MH-SR formulations.

## Introduction

Diabetes is a serious threat to human health, particularly in China. Globally, 537 million adults (20–79 years) are living with diabetes (1 in 10), and this number is predicted to rise to 643 million by 2030 and 783 million by 2045 [1]. In China, the estimated prevalence of diabetes in 2018 was 12.4% [95% confidence interval (CI), 11.8–13.0%] [2]. Metformin, a double guanidine drug, is the first-line treatment for type 2 diabetes [3,4].

Metformin is an amphoteric compound with pKa values of 2.8 and 11.5. The absolute oral bioavailability of metformin is 50–60%, and the gastrointestinal absorption is apparently complete within 6 h of ingestion. Metformin is rapidly distributed without any plasma-protein binding, and it undergoes renal excretion owing to its high hydrophilicity [5,6]. The most frequent adverse events (AEs) associated with metformin are gastrointestinal [7–9].

Compared with metformin hydrochloride tablets, metformin hydrochloride sustained-release (MH-SR) tablets are characterized by sustained drug release, long-lasting efficacy, decreased frequency of medication, fewer side effects and enhanced patient compliance [10].

Generic MH-SR tablets are crucial for the vast Chinese pharmaceutical market because it addresses the limited supply and high prices of the innovator MH-SR [11]. China joined the International Council for Harmonization of Technical Requirements for Pharmaceuticals for Human Use (ICH) in 2017 [12]. According to the requirements of the National Medical Products Administration (NMPA), generic drugs must be evaluated for consistency in quality and efficacy compared with the innovator drug [13].

The purpose of this study was to evaluate the bioequivalence and safety of generic MH-SR tablets (trade name: Boke^®^) developed by Beijing Wanhui Double-crane Pharmaceutical Co. Ltd., China and the innovator product, metformin hydrochloride extended-release (trade name: Glucophage^®^-XR) manufactured by Bristol-Myers Squibb Company, New York, NY, USA. The study was conducted under single-dose administration and fasting conditions in healthy Chinese volunteers.

## Materials and methods

### Study products

The study products were Boke^®^ (500 mg/tablet), the test preparation, and Glucophage^®^-XR (500 mg/tablet), the reference preparation. They were provided by the study sponsor, Beijing Wanhui Double-crane Pharmaceutical Co. Ltd., Beijing, China. Characteristics of the study products are presented in Table 1.

**Table 1.** Characteristics of the study products.

	Test product	Reference product
Ingredients	MH-SR	MH-XR
Dosage	500** **mg per tablet	500** **mg per tablet
Trade name	Boke^®^	Glucophage^®^-XR
Lot number	1160513	5B82400
Content	98.9%	97.3%
Manufacturing date	16 May 2016	01 February 2015
Expiry date	30 April 2019	31 January 2018
Manufacturer	Beijing Wanhui Double-crane Pharmaceutical Co., Ltd., China	Bristol-Myers Squibb Company, USA

### Study design

Two MH-SR formulations were evaluated for bioequivalence and safety in 48 healthy Chinese volunteers under fasting conditions. The study was designed as a single-centre, randomized, open-label, two-period, two-sequence crossover study, involving the administration of a single dose of 500 mg. The clinical trial was conducted at the Clinical Trial Centre of Beijing Shijitan Hospital, affiliated with the Capital Medical University, China, from 29 November 2017 to 16 April 2018. The study protocol and related documents were reviewed and approved by the Institutional Review Board of Beijing Shijitan Hospital (approval no. 2017Y94) on 16 October 2017. The clinical trial complied with the Declaration of Helsinki, Good Clinical Practice Guidelines of the ICH, Good Laboratory Practice, Pharmaceutical Administration Law, and other local laws and regulations. This clinical trial was registered in the Chinese Clinical Trial Registry (Registration No. CTR20171306). Written informed consent was obtained from all the participants prior to the study. All mandatory laboratory health and safety procedures have been complied with in the course of conducting any experimental work reported in this report.

### Participants

Participants were screened 1–7 d before administration. Demographic data, medical history (information provided by participants and then collected from the Hospital Information System by researchers), and smoking history of the volunteers were collected. The following examinations were conducted: vital signs (blood pressure, pulse, breathing and body temperature), 12-lead electrocardiogram (ECG), physical examination and clinical laboratory examination (routine urine and blood tests and blood biochemistry), serum virology tests (hepatitis B virus surface antigen, hepatitis C virus antibody and *Treponema pallidum* antibody), and human immunodeficiency virus antibody test. Additionally, women of childbearing age underwent a blood pregnancy test.

The study enrolled healthy Chinese adult volunteers aged 18 years and older with a body mass index of 19.0–28.0 kg/m^2^, male body weight ≥50 kg, and female body weight 0–28 kg. The inclusion criteria were as follows: individuals not addicted to drugs, alcohol or smoking and those without comorbid conditions, including cardiovascular, hepatic, renal, digestive tract, respiratory, neurological and metabolic abnormalities. The inclusion of the participants was based on the clinical judgement of the investigators, according to medical history and clinical examination. A negative pregnancy test prior to enrolment was an essential condition for including female participants, and they were required to use adequate contraception until 6 months after the second administration. The key exclusion criteria were as follows: consumption of any drug within 2 weeks before the clinical trial, a history of acupuncture syncope, difficulty in collecting venous blood, and blood loss or blood donation of >400 mL within 3 months before the trial.

Withdrawal criteria: The investigator had the right to withhold a participant from further dosing if the participant experienced AEs or symptoms or conditions listed in the exclusion criteria during the study or if the participants decided to drop out during the trial under any circumstances.

### Drug administration and blood sample collection

**Figure 1.**
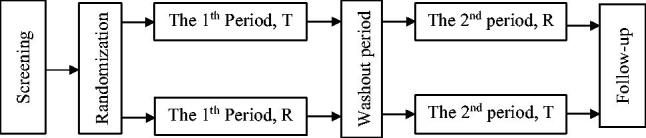
2-Crossover trial flow design. *Notes*: T: test preparation = Boke^®^; R: reference preparation = Glucophage^®^-XR. Washout period = 7 d.

The predesigned random allocation sequence was generated using SAS version 9.2 (SAS Institute, Cary, NC, USA) by a statistician, and the random seed number was saved for verification. Forty-eight healthy Chinese participants were randomly assigned to one of the two dosing-order subgroups, reference-test (R-T) and test-reference (T-R) at a 1:1 ratio, using a random allocation sequence. After overnight fasting for 10 h, each participant was administered a single dose of either preparation of MH-SR (T/R) at 500 mg with 240 mL of water, according to the study protocol. Lunch and dinner were served 4 and 10 h after drug administration, respectively (Figure 1).

For the metformin assay, venous blood samples (approximately 4 mL) were drawn from all participants through an indwelling cannula into tubes containing the anticoagulant K2-ethylenediaminetetraacetic acid, before administration (0 h; within 10 min before administration) and 1, 1.5, 2.5, 3, 4, 5, 6, 7, 8, 9, 10, 12, 15, 24, 36 and 48 h after administration. The blood samples were centrifuged at 2500 × *g* at 4 °C for 10 min, and the obtained plasma was transferred to freshly labelled tubes, kept frozen at −20 ± 10 °C for 1 h and subsequently at −70 ± 10 °C for 3 d, and stored until further analysis. After a washout period of 7 d, the formulation was cross-administered.

Medical surveillance was performed continuously throughout the trial. The vital signs of the participants were monitored during the screening period, before admission and departure from the hospital during each period, and 4, 8, 24 and 48 h after administration. The following safety checks were conducted before the participants left the research centre on the third day (D3) in the first period, when they returned to the hospital the day before the second dose, and before they left the research centre again at D10 in the second period: laboratory tests (including blood biochemistry, blood routine, urine routine and serum pregnancy test for women only) and physical examination. In addition to the above examinations, the participants were subjected to substance abuse tests and alcohol breath tests and were required to monitor their ECG before leaving the hospital after trial completion at D10 in the second period (or prior to withdrawal). Adverse events were closely observed during the trial, and the participants were asked to report any discomfort. Drug combination and non-drug treatment were recorded in detail during the trial. All AEs occurring during the trial were followed up until the disappearance of the AE, stabilization, or loss of follow-up.

### Bioanalytical methods

Quantitative analysis of metformin in the plasma was conducted on an LC-30AD liquid pump high-performance liquid chromatography (HPLC) system (AB Sciex, Foster City, USA), combined with an API 4000 Triple Quadrupole Mass Spectrometer (MS/MS) (Shimadzu, Kyoto, Japan). Metformin was extracted from the plasma using protein precipitation and concentration methods [14]. The plasma sample (100 μL) was added into a 96-well plate, followed by the addition of an internal standard solution (metformin-D_6_, 250 ng/mL) or 20 μL of methanol/aqueous solution (20:80 *v/v*). Methanol (500 μL) was added after vortex oscillation for 30 s (1000 × *g*), followed by vortex oscillation for 10 min (1000 × *g*) and centrifugation for 10 min (6000 × *g*). The resulting supernatant (5 μL) was injected into an HPLC-MS/MS system for analysis.

Method validation was performed according to the NMPA and Drug Administration Bioanalytical Method Validation Guidance. The linear range was defined as 1–1000 ng/mL, based on verification. The lower limit of quantification for metformin was 1 ng/mL, using a validated HPLC-MS/MS method. All the other results, such as selectivity, lower limit of quantitation, precision, accuracy, matrix effect, recovery and stability met the acceptance criteria.

Plasma metformin concentrations were analysed by Frontage Laboratories Inc. (Shanghai, China), according to the guidelines for bioequivalence published by the China Food and drug Administration (CFDA, renamed as NMPA in 2018).

### Pharmacokinetic analysis

Pharmacokinetic analysis was conducted only for samples from participants who had taken the trial drugs in two periods. Pharmacokinetic parameters were analysed using Certara Phoenix WinNonlin version 7.0 (Pharsight Corporation, Princeton, NJ, USA).

The blood concentration–time curve (C–T curve) was obtained by mapping the plasma metformin concentration at each time point. The peak concentration (*C*_max_) was obtained from this graph. The area under the curve (AUC) from time 0 to the time of the last measurable concentration (AUC_0→_*_t_*) was calculated using the linear trapezoidal rule. The AUC from time 0 to infinity (AUC_0→∝_) was obtained using the formula AUC_0→∝_ = AUC_0→_*_t_* + C*_t_*/*λ*_z_, where C*_t_* is the final measurable concentration and *λ*_z_ is the elimination rate constant, which is the slope of the terminal segment of the linear regression curve of the logarithmically transformed drug concentration values *vs.* time. Moreover, the time to peak concentration (*T*_max_) was obtained directly from the C–T curve. *T*_1/2_ is the elimination half-life, calculated as 0.693/*λ*_z_. Log-transformed primary pharmacokinetic parameters were tested using analysis of variance (ANOVA). The results of the mixed effects model included sequence, period and preparation factors [11,13,15].

Pharmacokinetic bioequivalence analysis of MH-SR and Glucophage^®^-XR was conducted using the geometric mean ratio (GMR), 90% CI, and two-sided *t*-test. Pharmacokinetic bioequivalence was established between MH-SR and Glucophage^®^-XR if the 90% CIs of the main pharmacokinetic parameters were within the equivalent interval (80.00–125.00%).

### Safety assessment

We statistically analysed data from all randomized participants who were administered the trial drug and who had safety records. We monitored AEs, serious AEs (SAEs), concomitant medication, non-drug therapies, abnormal clinical manifestations, changes in laboratory results (routine blood, urine, stool and blood biochemistry), and ECG to ensure the safety of the clinical trial. All participants with AEs, whether considered drug-related or not, were regularly monitored if feasible until recovery, stability, or irreversible damage.

All AEs were assessed using the Common Terminology Criteria for Adverse Events version 4.03. The degree of correlation between AEs and drug administration was classified into the following five levels, according to the judgement standard of causality between drugs and AEs: definitely unrelated, unlikely related, possibly related, probably related and definitely related. Events classified as possibly related, probably related and definitely related were listed as adverse drug reactions (ADRs).

### Statistical analysis

According to previous clinical studies, the mean intra-individual coefficient of variation (CV%) for AUC of MH-SR tablets is 22.2% [16]. The GMR of the test and reference preparations (T/R) was set as 1.1, the power was 0.80 (1 – *β* = 0.80), and the bilateral significance level was .05 (*α* = 0.05). It was estimated that a minimum sample size of 38 was required to prove the bioequivalence of MH-SR. Considering the 20% risk of dropout, a sample size of 48 participants was considered in the trial [17].

The normality of the measured data was tested using the KS method. Data with normal distribution were represented as the mean ± standard deviation (x±s), and the difference between the two groups was tested using the *t*-test. Data with a non-normal distribution were represented as the quartile (25 and 75%), and the difference between the two groups was tested using the Wilcoxon rank-sum test. Count data were represented as numbers and percentages (*n*, %), and the difference between the two groups was tested using the chi-square test.

The main pharmacokinetic parameters were logarithmically transformed before statistical analysis since they are usually distributed log-normally [11,13]. Any difference between the two related parameters was deemed statistically significant at *p* ≤ .05. Statistical analyses were performed using SAS version 9.2.

## Results

### Participants

**Figure 2.**
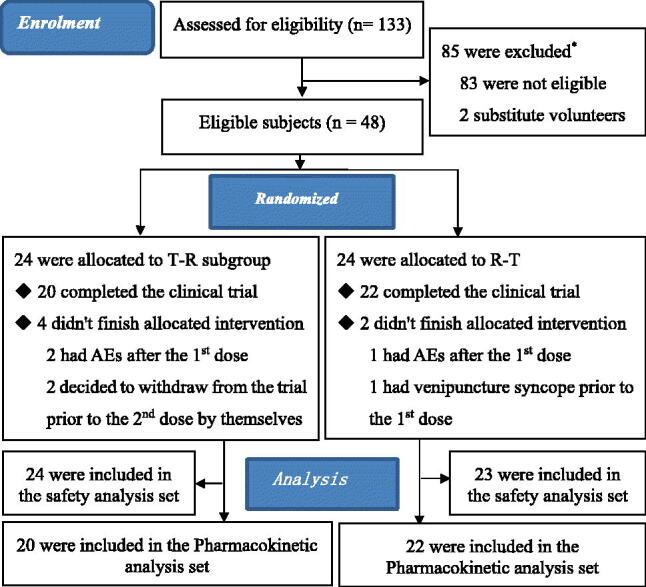
Flow diagram of screening participants for the clinical trial. *Notes*: T: test preparation = Boke^®^; R: Reference preparation = Glucophage^®^-XR; AEs: adverse events. *The reasons of ineligibility: single A (60), single B (11), single D (2), single E (7), combined A + C (1), combined A + D (1) and combined D + E (1). (A) Abnormality in physical examination, vital sign examination, laboratory examination and electrocardiogram; (B) Previous digestive disease or surgery that may interfere with drug absorption; (C) Hepatitis B surface antigen test positive or active hepatitis B; (D) Use of any other drugs in the two weeks prior to the start of the study; (E): The investigator determined that the subject could not complete the study or otherwise believed that the subject’s participation in the study might cause harm. ^#^The two substitute volunteers were used for replacement volunteers who were unable to participate in the trial for various reasons before randomization.

A total of 133 participants were selected for the fasting test, of which 85 (63.9%) failed and 48 (36.1%) were randomly allocated to the T-R or R-T subgroups. Among the included participants, 36 (75.00%, 36/48) were men (Figure 2). Han ethnicity accounted for 97.92% (47/48). The age of the participants was 27.9 ± 7.6 years (range: 18–46 years); body weight, 65.2 ± 8.0 kg (range: 52.9–83.7 kg); height, 168.7 ± 7.0 cm (range: 153.8–186.1 cm); and body mass index, 22.9 ± 2.3 kg/m^2^ (range: 19.1–27.5 kg/m^2^). There were no significant differences in the demographic characteristics between the T-R and R-T subgroups (Table 2).

**Table 2.** The demographic and baseline characteristics of subjects in the clinical trials.

Characteristics	T-R	R-T	Statistic	*p* Value
Number (*n*)	24	24	–	–
Age (years)	28.3** **±** **7.9	27.5** **±** **7.3	−0.36^^^	.721
Gender, male (*n*, %)	17 (70.8)	19 (79.2)	0.44^#^	.505
The Han Ethnicity (*n*, %)	24 (100.0)	23 (95.8)	–	.999*
Height (cm)	169.5** **±** **7.2	168.0** **±** **6.8	−0.75^^^	.460
Body weight (kg)	66.0** **±** **8.8	64.3** **±** **7.3	−0.73^^^	.467
Body mass index (kg/m^2^)	23.0** **±** **2.5	22.8** **±** **2.2	−0.24^^^	.812

*Notes*: ^^^*t* value; ^#^Chi square value; *Fisher’s exact test; T: test preparation = generic metformin hydrochloride sustained-release tablets; R: reference preparation = Glucophage-XR.

Forty-two participants completed the trial, while the remaining six participants withdrew from the trial. One participant experienced venipuncture syncope prior to the first period of administration, two decided by themselves to withdraw from the trial prior to the second dose administration, and the other three reported AEs following the first dose (Supplementary Table S1). The safety analysis set included 47 participants; 42 participants were a part of the pharmacokinetic analysis set (Figure 2).

### Bioequivalence assessment

Table 3 shows the main pharmacokinetic parameters of MH-SR (500 mg) and Glucophage^®^-XR (500 mg) in the study participants. No significant difference was observed in the main pharmacokinetic parameters between MH-SR and Glucophage^®^-XR (*p* = .81, .76 and .61 for AUC_0→_*_t_*, AUC_0→∝_ and *C*_max_, respectively).

**Table 3.** Comparison of arithmetic mean of generic metformin hydrochloride sustained-release tablets (MH-SR, 0.5** **g) and Glucophage-XR (0.5** **g) in healthy Chinese volunteers under single-dose administration and fasting conditions (*n*** **=** **42).

Pharmacokinetic parameter	T (MH-SR)	R (Glucophage-XR)	*t* Value	*p* Value
AUC_0→_*_t_* (h·mg·L^−1^)	5080.60** **±** **1132.68	4999.6** **±** **1254.62	−0.24	.81
AUC_0→∝_ (hr·mg·L^−1^)	5203.80** **±** **1255.33	5099.00** **±** **1231.00	−0.31	.76
*C*_max_ (mg·L^−1^)	724.14** **±** **216.43	697.54** **±** **199.03	−0.52	.61
*T_max_ (h)	4.00 (1.50, 5.00)	4.00 (1.50, 5.00)	0.67	.50
*t*_1/2_ (h)	10.38** **±** **7.10	10.23** **±** **6.79	−0.06	.96
λ_z_ (1/h)	0.09** **±** **0.03	0.08** **±** **0.03	−0.15	.88

*Notes*: *a non-normal distribution data was denoted by the quartile and tested by chi-square test Wilcoxon rank sum test.

T: test preparation; R: reference preparation.

Table 4 shows the bioequivalence analysis results for MH-SR and Glucophage^®^-XR in the study participants under single-dose administration and fasting conditions. The GMR ratio (T/R) of AUC_0→_*_t_*, AUC_0→∝_ and *C*_max_ of MH-SR and Glucophage^®^-XR were 102.68, 102.53 and 103.81%, respectively; the 90% CIs were 97.36–108.30, 97.26–108.09 and 96.76–111.37%, respectively. Since the 90% CIs of the main pharmacokinetic parameters were within the equivalent interval (80.00–125.00%), we conclude that the test and reference formulations were bioequivalent under single-dose administration and fasting conditions.

**Table 4.** Bioequivalence analysis of generic metformin hydrochloride sustained-release tablets (T, 0.5** **g) and Glucophage-XR (R, 0.5** **g) in healthy Chinese volunteers (*n*** **=** **42) under single-dose administration and fasting conditions.

Pharmacokinetic parameter	Geometric mean value and ratio			90% CI (%)	COV (%)	Power (%)
	T	R	T/R (%)			
AUC_0→_*_t_* (hr·ng/mL)	4960.65	4831.00	102.68	97.36–108.30	14.54	100.0
AUC_0→∞_ (hr·ng/mL)	5070.54	4945.31	102.53	97.26–108.09	14.42	100.0
*C*_max_ (ng/mL)	694.42	668.94	103.81	96.76–111.37	19.29	99.7

*Note*: T: test preparation; R: reference preparation; COV: coefficient of variation here means intra-individual variation.

Power** **=** **1 – *β*, type II error.

**Figure 3.**
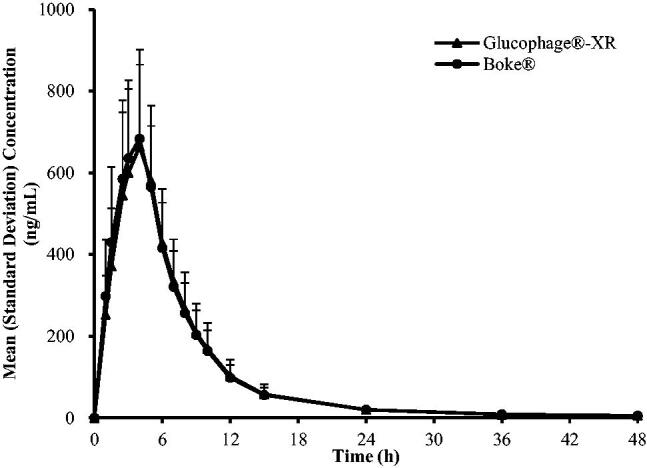
Mean (*n*** **=** **42) plasma concentration-time curves of Boke^® ^and Glucophage^®^-XR in healthy Chinese volunteers under single-dose administration and fasting conditions (linear plot).

**Figure 4.**
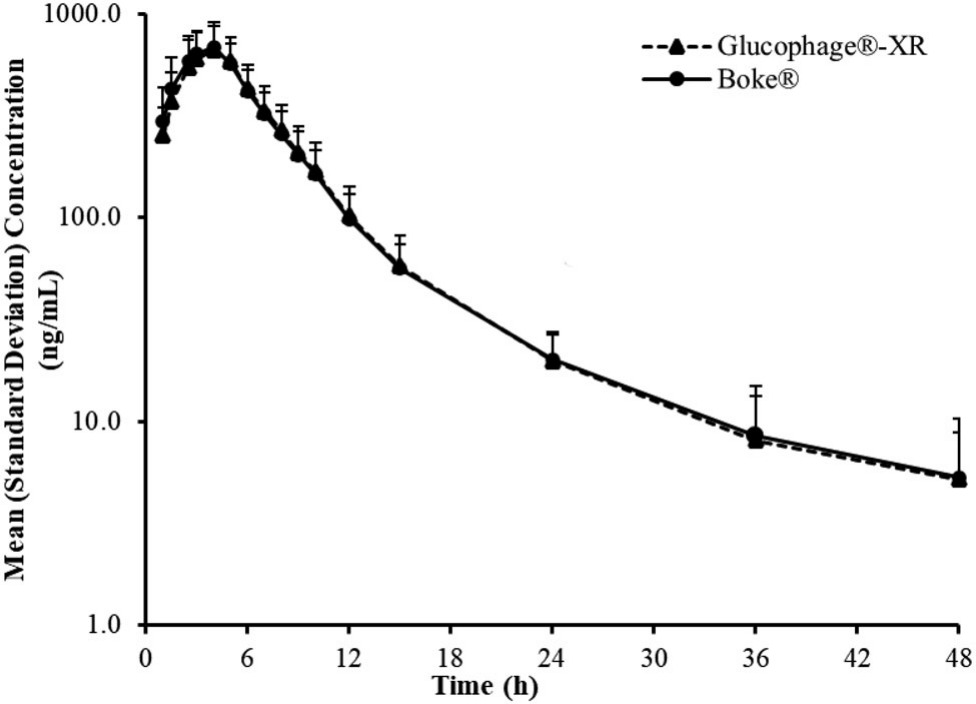
Mean (*n* = 42) plasma concentration-time curves of Boke^®^ and Glucophage-XR in healthy Chinese volunteers under single-dose administration and fasting conditions (semilogarithmic plot).

The C–T curves of Boke^®^ and Glucophage^®^-XR were in good agreement (Figures 3 and 4).

Additionally, no significant differences were observed between the main parameters of the test and reference product as identified by ANOVA (*p* = .17, .75 and .38 for sequence, periods and formulations, respectively) (Table 5). The results demonstrated that the mixed effects model included sequence, period and preparation factors that did not interfere with the conclusions of the study.

**Table 5.** Results (*p* values) of ANOVA test of bioequivalence between generic metformin hydrochloride sustained-release tablets (T, 0.5** **g) and Glucophage-XR (R, 0.5** **g) in healthy Chinese volunteers (*n*** **=** **42) under single-dose administration and fasting conditions.

Influence factor	lnAUC_0→_*_t_*	lnAUC_0→∞_	lnC_max_
Sequence	0.46	0.56	0.17
Periods	0.29	0.54	0.75
Formulations	0.41	0.43	0.38

**Figure 5.**
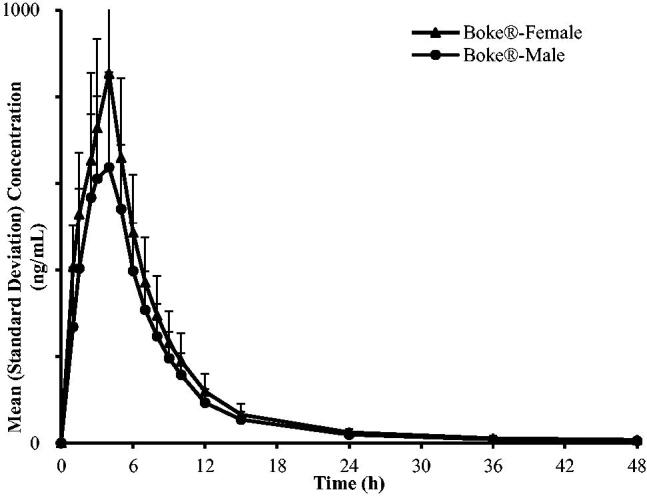
Mean plasma concentration-time curves of Boke^®^ categorize by gender of healthy Chinese volunteers under single-dose administration and fasting conditions (linear plot).

**Figure 6.**
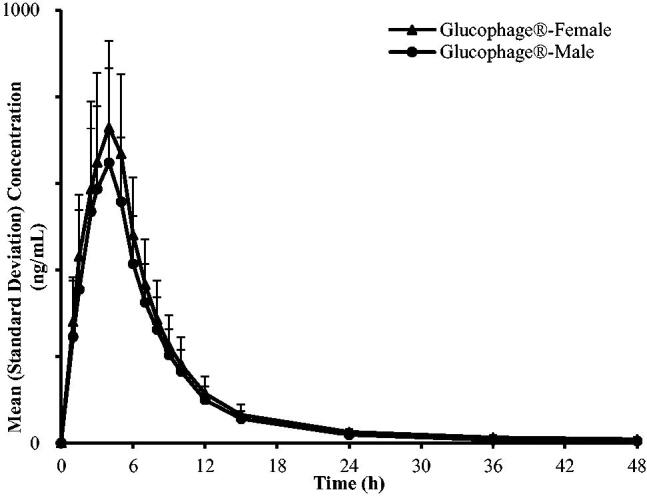
Mean plasma concentration-time curves of Glucophage^®^-XR categorize by gender of healthy Chinese volunteers under single-dose administration and fasting conditions (linear plot).

Post-hoc analysis revealed that the AUC_0→_*_t_*, AUC_0→∝_ and *C*_max_ of Boke^®^ and Glucophage^®^-XR in females were higher than the corresponding values in males (6037.05 ± 1264.46 *vs.* 4819.72 ± 916.47 ng/mL, 6276.50 ± 1628.88  *vs*. 4911.17 ± 913.46 ng/mL, 893.30 ± 273.65  vs. 678.02 ± 166.92 ng/mL, respectively, for Boke^®^ and 5621.24 ± 866.29 *vs.* 4830.32 ± 1271.17 ng/mL, 5803.62 ± 838.00  *vs.* 4906.80 ± 1232.48 ng/mL, 744.88 ± 189.84 vs. 684.62 ± 196.48 ng/mL, respectively, for Glucophage^®^-XR). Figures 5 and 6 show that the C–T curves of Boke^®^ and Glucophage^®^-XR are categorized by the gender of the participants.

### Safety and tolerability

In total, 41 AEs occurred in 19 participants. Of the 46 participants who were administered the test drug, 10 participants (21.74%, 10/46) showed 23 ADRs. Of the 43 participants who were administered the reference drug, Glucophage^®^-XR, 12 participants (27.91%, 12/43) showed 15 ADRs. No significant differences in ADRs were identified between the test and reference products (21.74 vs. 27.91%, *p* = .500) (Table 6).

**Table 6.** Adverse events of generic metformin hydrochloride sustained-release tablets (0.5** **g, T) and Glucophage-XR (0.5** **g, R).

Adverse events	T (*n*** **=** **46)		R (*n*** **=** **43)		*χ* ^2^	*p* Value
	AEs (*N*)	Cases (*n*, %)	AEs (*N*)	Cases (*n*, %)		
Metabolic or nutritional diseases	3	3 (6.5)	5	5 (11.6)	–	.475*
Hypertriglyceridemia	1	1 (2.2)	3	3 (7.0)	–	.350*
Hyperuricemia	2	2 (4.3)	2	2 (4.7)	–	.999*
Infectious diseases	2	2 (4.3)	0	0 (0.0)	–	.495*
Upper respiratory tract infection	2	2 (4.3)	0	0 (0.0)	–	.495*
All kinds of abnormal inspection	12	5 (10.9)	6	5 (11.6)	–	.999*
γ-Glutamyl-transferase (^↑^)	0	0 (0.0)	1	1 (2.3)	–	.483*
Alanine aminotransferase (^↑^)	0	0 (0.0)	1	1 (2.3)	–	.483*
Conjugated bilirubin (^↑^)	4	4 (8.7)	2	2 (4.7)	–	.678*
Urine ketone bodies (^+^)	1	1 (2.2)	0	0 (0.0)	–	.999*
Blood in the urine	1	1 (2.2)	0	0 (0.0)	–	.999*
Blood bilirubin (^↑^)	3	3 (6.5)	0	0 (0.0)	–	.242*
Blood unbound bilirubin (^↑^)	3	3 (6.5)	0	0 (0.0)	–	.242*
Serum creatine phosphokinase (^↑^)	0	0 (0.0)	1	1 (2.3)	–	.483*
Neutrophil count (^↑^)	0	0 (0.0)	1	1 (2.3)	–	.483*
Gastrointestinal diseases	6	4 (8.7)	4	4 (9.3)	–	.999*
Abdominal pain	3	3 (6.5)	0	0 (0.0)	–	.242*
Diarrhoea	3	3 (6.5)	3	3 (7.0)	–	.999*
Abdominal distension	0	0 (0.0)	1	1 (2.3)	–	.483*
Total	23	10 (21.7)	15	12 (27.9)	0.454	.500

*Note*: *Fisher exact test; ↑increasing; +positive.

T: test preparation; R: reference preparation.

Among the 23 ADRs during the administration of MH-SR, 15 ADRs (65.22%, 15/23) and 8 ADRs (34.78%, 8/23) were of common terminology criteria for adverse events (CTCAE) Grade 1 and Grade 2, respectively. Among the 15 ADRs during the administration of Glucophage^®^-XR, 13 ADRs (86.67%, 13/15) and 2 ADRs (13.33%, 2/15) were of CTCAE Grade 1 and Grade 3, respectively. No significant differences in ADR severity were identified between MH-SR and Glucophage^®^-XR (*p* = .26, Fisher’s Exact Test). The outcome of all the above-mentioned ADRs was “recovered/resolved”. The details of all AEs that occurred during the trial are listed in Supplementary Table S2.

## Discussion

The main goal of this study is to support an increase in the supply of MH-SR in China by proving the efficacy and safety of a generic alternative. This trial was performed according to the last national regulation, a strict standard [18]. The innovator drug, Glucophage^®^-XR (500 mg), was used as a reference. HPLC-MS/MS, which is currently widely used for bioequivalence evaluation, was used in this clinical trial [13].

Zhou et al. [19] studied the bioequivalence of MH-SR tablets and Glucophage^®^-XR (500 mg) as a reference preparation under fed states. HPLC-MS/MS was used for testing, and 19 blood sampling points within 0–36 h were selected. The following data were obtained for the test and reference preparations in Zhou et al. study and our study under fed states, respectively: AUC_0→36_ = 6.56 ± 0.81 (range 5.31–7.80) h·mg·L^−1^ and 6.75 ± 1.22 (range 4.89–8.60) h·mg·L^−1^; AUC_0→∝_ = 6.63 ± 0.82 (range 5.34–7.86) h·mg·L^−1^ and 6.83 ± 1.22 (range 4.93–8.70) h·mg·L^−1^; AUC_0→48_ = 7.33 ± 1.65 and 7.00 ± 1.89h·mg·L^−1^; and AUC_0→∝_ = 7.39 ± 1.67 and 7.06 ± 1.91h·mg·L^−1^. No significant differences were observed between the main pharmacokinetic parameters. We previously reported the bioequivalence of the generic MH-SR produced by Guangdong Sinocorp Pharmaceutical Co. Ltd., Guangdong, China and the innovator product Glucophage^®^-XR in healthy Chinese participants, with similar results [11]. The values of the main pharmacokinetic parameters in the above studies were different from that in this study because of differences between postprandial and fasting conditions. A thorough literature search revealed more reports on the bioequivalence of MH-SR under postprandial conditions than that under fasting conditions.

According to the requirements of NMPA, bioequivalence tests of MH-SR tablets were conducted successively under fasting and postprandial conditions. Here, the results of bioequivalence trials under fasting conditions are reported. Compared with the results of the postprandial trial, AUC_0→_*_t_* and AUC_0→∝_ are lower (4960.65 vs. 7148.53 h·ng/mL and 5070.54 vs. 7205.59h·ng/mL), while *C*_max_ is slightly higher (694.42 vs. 671.57 ng/mL) under fasting conditions.

The instruction manual of Glucophage^®^ and Glucophage^®^-XR specifies that metformin pharmacokinetic parameters did not differ significantly between normal subjects and patients with type 2 diabetes mellitus when analysed according to gender (males = 19 and females = 16) [20]. However, the results of this study indicate that gender could affect the pharmacokinetics of metformin. Animal experiments have shown that gender-related differences were observed in the pharmacokinetics of metformin when administered at relatively high doses; the plasma concentration of metformin in female rats was significantly increased compared to that in male rats at 2 h after oral administration [21].

Metformin has been used in clinical practice for more than 60 years, and sufficient data are available to prove its safety [22]. No SAEs or unexpected AEs occurred in healthy Chinese participants who were administered MH-SR or Glucophage^®^-XR in this clinical trial. The number of ADRs in this study was limited. All types of abnormal inspection occurred most frequently during this clinical trial, followed by gastrointestinal, metabolic or nutritional diseases. In the instruction manual of Glucophage^®^ and Glucophage^®^-XR 2020, the most common adverse reactions reported in the GLUCOPHAGE XR (*n* = 781) were diarrhoea (10%) and nausea (7%). This result is different from that reported in this study (*n* = 48); this inconsistency may be related to the small sample size of this study.

According to the BE guidelines for metformin hydrochloride tablets by the FDA, the drug products should be administered with 240 mL of a 20% glucose water solution, followed by administration of 60 mL of the glucose solution every 15 min for up to 4 h [15]. However, metformin does not generally cause hypoglycaemia in healthy participants since it increases endogenous glucose production in non-diabetic individuals [23]. No event of hypoglycaemia occurred even though no sugar was given in this fasting condition. Both MH-SR and Glucophage^®^-XR were safe and well tolerated.

However, this study had some limitations. First, the study was designed as a single-centred clinical trial with a limited sample size. The small sample size did not reveal all ADRs of the MH-SR, such as vitamin B_12_ deficiency and lactic acidosis (Supplementary, Instruction manual of Boke^®^). Fortunately, there were no significant differences in the ADRs between the two MH-SR formulations. The absence of all ADRs that occurred in the study affected the judgement of the results. Moreover, China is a multi-ethnic country, and most of the participants in this study were Han. The impact of ethnicity on the differences between the two preparations was not considered in this study. However, this limitation could be offset by the results of a previous study, which demonstrated that the ADRs of the MH-SR formulation do not differ significantly among different ethnic groups [15].

## Conclusions

Generic MH-SR tablets (Beijing Wanhui Double-crane Pharmaceutical Co. Ltd., China) and innovator MH-SR tablets (Glucophage^®^-XR, Bristol-Myers Squibb Company, New York, NY, USA) were bioequivalent and safe in healthy Chinese volunteers under single-dose administration and fasting conditions.

## Supplementary Material

Supplemental Material

## Data Availability

Data sharing is not applicable to this article as no new data were created or analysed in this study.
