# Supplementary material for: Bioequivalence and safety evaluation of two preparations of metformin hydrochloride sustained-release tablets (Boke® and Glucophage®-XR) in healthy Chinese volunteers: a randomized phase I clinical trial
Source: Ann Med. 2022 Sep 22;54(1):2617–26. doi: 10.1080/07853890.2022.2125574 (PMC9521607; doi:10.1080/07853890.2022.2125574)
Supplement: Supplemental Material [file IANN_A_2125574_SM9308.docx]

Bioequivalence and safety evaluation of two preparations of metformin hydrochloride sustained-release tablets (Boke^®^ and Glucophage^®^-XR) in healthy Chinese volunteers: a randomised phase I clinical trial

Ming-Li Sun^a^, Chen Liu^a^, Hai-Hong Bai^a^, Ya-Li Wei^a^, Wei Zhang^a^, Hui-Juan Liu^a^, Yin-Juan Li^a^, Long Liu^a^, Yu Wang^a^, Yuan-Xv Tong^a^, Qian Gao^b^, Qian-Ying Liu^c^, and Xinghe Wang^a^*

Supplementary

Content

Table S1 AE occurred in 3 participants who withdrew due to AE .................................................................................................................................1

Table S2 Detail of all adverse events during the trail ....................................................................................................................................................3

Drug specifications of Boke^®^ .................................................................................................................................................................................................................5

Table S1. AE occurred in 3 participants who withdrew due to AE

| Serial number | The name of the AE | Relationship with drugs | Time of occurrence (after oral) | Duration | Severity (Level) | Value | Intervention | Reasons for withdrawal of participants |
| --- | --- | --- | --- | --- | --- | --- | --- | --- |
| 1003 | Diarrhea | Possibly related | 6 h 6 min | 10 min | 1 | NA | No | AE did not return to normal or NCS before the 2^nd^ period of administration. The participant did not meet the inclusion and exclusion criteria, and, thus, was withdrawn from the trial. |
|  | Alanine aminotransferase (↑) | Possibly related | 6 D 5 min | 14D 29min | 3 | 109→608→339→73→25 U/L | Oral silybin capsule 70 mg/tablet, tid |  |
|  | γ-glutamyl-transferase (↑) | Possibly related | 6 D 5 min | 14D 29min | 3 | 6→261→173→84→47 U/L (NCS) |  |  |
|  | Gallbladder silt stone | Unlikely related | 8 D 51 min | UK | NA | NA | No |  |
|  | Vaginitis | Unlikely related | 8D1h40min | UK | NA | NA | No |  |
| 1015 | Abdominal pain | Possibly related | 16 h 32min | 6 h | 1 | NA | No | The last blood sample for bilirubin exam was collected 1 h 13 min before administration of the 2^nd^ period. Although the results returned to normal, the report was obtained after the scheduled time for the 2^nd^ administration time, and, thus, the participant was withdrawn. |
|  | Diarrhea | Possibly related | 2D11h32min | 8 min | 1 | NA | No |  |
|  | Blood bilirubin ↑ | Possibly related | 2 D | 4D22h 47min | 2 | Screen time 23.4 (NCS) → P1: 25.1 → 27.8 → 31.1 → 35 → 23.7 umol/L (NCS) | No |  |
|  | Blood unbound bilirubin (↑) | Possibly related | 2 D | 4D 8h  26min | 2 | Screen time 7.5 (NCS) → P1:8.2 →9.0→ 8.0 → 11.2 → 7.9 umol/L (NCS) | No |  |
|  | Conjugated bilirubin (↑) | Possibly related | 2 D | 3D22h 47min | 2 | Screen time15.9(NCS)→P1:16.9→18.8→23.1 →23.8→15.8 umol/L (NCS) | No |  |
|  | Urine ketone bodies (+) | Possibly related | 2D 17min | 4D10h 45min | 1 | 1+→1+→3+→1+→- | No |  |
| Blood unbound bilirubin (↑) | Blood unbound bilirubin (↑) | Possibly related | 2 h 54 min | 10 min | 1 | NA | No | The participant was withdrawn from the 2^nd^ period of the trial because of not meeting the inclusion and exclusion criteria due to drug use. |
|  | Blood unbound bilirubin (↑) | Possibly related | 6D20h44min | 2D 3h5min | 2 | White blood cell 12.46×109/L CS; Review 4.40 Normal  Blood lymphocyte percentage (LY%) 7.3% CS; Review 42.0% normal  Blood monocytes (MO#) 1.1×10^9^/L CS; Review 0.48 normal  Blood neutrophils (NE#) 10.4×10^9^/L CS; Review 2.0 Normal | 2017-12-13: Oral volavol (cefuroxime axetil tablets) 0.5G bid  2017-12-13~14: Oral Tylenol (acetaminophen sustained release tablet) 0.65g  2017-12-13~14: Oral cefdinir dispersible tablets 0.1g 3 times/day  2017-12-13~14: Oral vitamin C yinqiao tablets 2 tablets tid  2017-12-13~14: Oral lotus Qingwen capsule 4 tablets tid |  |
|  | Blood unbound bilirubin (↑) | Possibly related | 2 D | 2D23h 19min | 1 | 8.8→6.8 μmol/L | No |  |

Table S2. Detail of all adverse events during the trail

| Serial number | Time of administration | Adverse events | Severity | Date and time of occurrence/end | Drug | Relationship with drugs |
| --- | --- | --- | --- | --- | --- | --- |
| 1003 | 2017-12-06 08:04 | Diarrhea | 1 | 2017-12-06 14:10/2017-12-06 14:20 | R | probably related |
|  | 2017-12-06 08:04 | Alanine aminotransferase (↑) | 3 | 2017-12-12 08:09/2017-12-26 08:38 | R | probably related |
|  | 2017-12-06 08:04 | γ-glutamyl-transferase (↑) | 3 | 2017-12-12 08:09/2017-12-26 08:38 | R | probably related |
|  | 2017-12-06 08:04 | Gallbladder silt stone | 1 | 2017-12-14 08:55/ UK | R | unlikely related |
|  | 2017-12-06 08:04 | Vaginitis | 1 | 2017-12-14 09:44/ UK | R | unlikely related |
| 1004 | 2017-12-13 08:06 | Hypertriglyceridemia | 1 | 2017-12-15 08:06/2017-12-18 08:20 | R | probably related |
| 1006 | 2017-12-06 08:10 | Blood unbound bilirubin (↑) | 1 | 2017-12-08 08:10/2017-12-11 08:33 | R | probably related |
|  | 2017-12-13 08:10 | Blood bilirubin (↑) | 1 | 2017-12-15 08:10/2017-12-18 08:02 | T | probably related |
|  | 2017-12-13 08:10 | Blood unbound bilirubin (↑) | 1 | 2017-12-15 08:10/2017-12-18 08:02 | T | probably related |
|  | 2017-12-13 08:10 | Conjugated bilirubin (↑) | 1 | 2017-12-15 08:10/2017-12-18 08:02 | T | probably related |
| 1008 | 2017-12-06 08:14 | Hypertriglyceridemia | 1 | 2017-12-08 08:14/2017-12-11 08:35 | R | probably related |
|  | 2017-12-06 08:14 | Serum creatine phosphokinase (↑) | 1 | 2017-12-08 08:14/2017-12-11 08:35 | R | probably related |
| 1009 | 2017-12-06 08:16 | Hyperuricemia | 1 | 2017-12-08 08:16/2017-12-11 08:36 | R | probably related |
| 1015 | 2017-12-06 08:28 | Abdominal pain | 1 | 2017-12-07 01:00/2017-12-10 07:00 | T | probably related |
|  | 2017-12-06 08:28 | Diarrhea | 1 | 2017-12-08 20:00/2017-12-08 20:08 | T | probably related |
|  | 2017-12-06 08:28 | Blood bilirubin (↑) | 2 | 2017-12-08 08:28/2017-12-13 07:15 | T | probably related |
|  | 2017-12-06 08:28 | Blood unbound bilirubin (↑) | 2 | 2017-12-08 08:28/2017-12-12 16:54 | T | probably related |
|  | 2017-12-06 08:28 | Conjugated bilirubin (↑) | 2 | 2017-12-08 08:28/2017-12-13 07:15 | T | probably related |
|  | 2017-12-06 08:28 | Urine ketone bodies (+) | 1 | 2017-12-08 08:45/2017-12-12 19:30 | T | probably related |
| 1016 | 2017-12-06 08:30 | Diarrhea | 1 | 2017-12-10 15:00/2017-12-10 15:10 | R | probably related |
|  | 2017-12-13 08:30 | Abdominal pain | 1 | 2017-12-13 18:58/2017-12-13 20:10 | T | probably related |
|  | 2017-12-13 08:30 | Diarrhea | 1 | 2017-12-13 19:58/2017-12-13 20:10 | T | probably related |
| 1017 | 2017-12-06 08:32 | Diarrhea | 1 | 2017-12-06 14:35/2017-12-06 14:38 | T | probably related |
|  | 2017-12-06 08:32 | Blood in the urine | 1 | 2017-12-08 08:13/2017-12-12 09:26 | T | probably related |
| 1027 | 2017-12-06 08:52 | Hyperuricemia | 1 | 2017-12-08 08:52/2017-12-11 08:38 | T | probably related |
| 1029 | 2017-12-06 08:56 | Diarrhea | 1 | 2017-12-06 13:21/2017-12-06 13:27 | R | probably related |
| 1033 | 2017-12-06 09:04 | Hypertriglyceridemia | 1 | 2017-12-08 09:04/2017-12-11 08:41 | R | probably related |
| 1034 | 2017-12-06 09:06 | Hypertriglyceridemia | 2 | 2017-12-08 09:06/2017-12-11 08:43 | T | probably related |
| 1035 | 2017-12-13 09:08 | Blood unbound bilirubin (↑) | 1 | 207-12-15/09:08 - 2017-12-18/08:05 | R | probably related |
| 1036 | 2017-12-13 09:10 | Blood unbound bilirubin (↑) | 2 | 2017-12-15 09:10/2018-01-12 11:15 | T | probably related |
|  | 2017-12-13 09:10 | Conjugated bilirubin (↑) | 2 | 2017-12-15 09:10/2018-01-12 11:15 | T | probably related |
|  | 2017-12-13 09:10 | Blood bilirubin (↑) | 2 | 2017-12-15 09:10/2018-01-12 11:15 | T | probably related |
|  | 2017-12-13 09:10 | Upper respiratory tract infection | 3 | 2018-01-01 15:00/2018-01-07 11:00 | T | unlikely related |
| 1040 | 2017-12-13 09:18 | Neutrophil count (↑) | 1 | 2017-12-15 14:05/2017-12-18 09:41 | R | probably related |
| 1043 | 2017-12-13 09:24 | Upper respiratory tract infection | 1 | 2017-12-15 07:00/2017-12-20 08:50 | T | probably related |
| 1044 | 2017-12-06 09:26 | Abdominal pain | 1 | 2017-12-06 12:20/2017-12-06 12:30 | T | probably related |
|  | 2017-12-06 09:26 | Upper respiratory tract infection | 2 | 2017-12-13 06:10/2017-12-15 09:15 | T | probably related |
|  | 2017-12-06 09:26 | Blood unbound bilirubin (↑) | 1 | 2017-12-08 09:26/2017-12-11 08:45 | T | probably related |
| 1046 | 2017-12-13 09:30 | Abdominal distension | 1 | 2017-12-13 11:30/2017-12-13 13:58 | R | probably related |
| 1047 | 2017-12-06 09:32 | Hyperuricemia | 1 | 2017-12-08 09:32/2017-12-12 16:48 | T | probably related |
|  | 2017-12-13 09:32 | Hyperuricemia | 1 | 2017-12-15 09:32/2017-12-18 08:07 | R | probably related |

Drug specifications of Boke^®^
